# Economic Benefits from the Use of Mass Trapping in the Management of Diamondback Moth, *Plutella xylostella*, in Central America

**DOI:** 10.3390/insects14020149

**Published:** 2023-02-01

**Authors:** Francisco Gonzalez, Carlos Rodríguez, Cam Oehlschlager

**Affiliations:** ChemTica Internacional S.A., Santo Domingo P.O. Box 640-3100, Costa Rica

**Keywords:** diamondback moth, mass trapping, pheromone, action threshold, monitoring

## Abstract

**Simple Summary:**

Introduction of monitoring of male diamondback moth using lures emitting female sex pheromone allowed farmers to determine the populations of adult DBM in cabbages in several cabbage farms in Costa Rica and Nicaragua. Farmers were encouraged to use trap capture data to trigger insecticide applications as an alternative to their normal practice of calendarized insecticide applications. Those farmers with the highest insecticide application rates against DBM were the most receptive to the idea of using pheromone-baited traps and reduced their spray regime by the largest proportion. Reduced spray schedules do not increase damage but can increase profits for farmers.

**Abstract:**

The diamondback moth, *Plutella xylostella* (L.), is a worldwide pest of brassica crops, resistant to a large number of insecticides. As an alternative, the use of pheromone-baited traps has been proposed but farmers are yet to be convinced. In the present study, we aimed to validate the benefits of the use of pheromone-baited traps for monitoring and mass trapping in cabbage production in Central America as means of Integrated Pest Management (IPM) in comparison to calendarized insecticide sprays, which are the farmers’ current practices (FCP). Mass trapping was established in nine selected plots of cabbage in Costa Rica and Nicaragua. Average captures of males/trap/night, plant damage and net profits of these IPM plots were compared to simultaneously evaluated or historically reported FCP plots. The results indicate that in Costa Rica, trap captures did not justify the application of insecticides and average net profits increased by more than 11% when the trapping methods were implemented. In Nicaragua, IPM plots were able to reduce insecticide applications to one third of those in FCP plots. These results confirm the economic and environmental benefits of pheromone-based management of DBM in Central America.

## 1. Introduction

Brassicaceae are worldwide components of human diets. In 2020, cabbage accounted for a harvested area of approximately 3,395,300 hectares, which yielded a total of 105,069,400 tons [1]. The economics of Brassicaceae cropping are significantly impacted by the diamondback moth (DBM), *Plutella xylostella* (L.) (Lepidoptera: Plutellidae). It has been estimated that the worldwide cost of managing *P. xylostella* in vegetable crops is USD 4–5 billion annually [2,3]. China, for example, is the principal grower of cabbages (*Brassica oleracea* var. *capitata*). It has seen a disproportionate increase in Brassica crop production and, consequently, the costs of DBM management have reached the outstanding value of approximately USD 0.77 billion per year [4]. 

The high costs of managing DBM are rooted in the biology of the pest and the strategies used for its control. This insect has a short life cycle and a wide range of hosts. It is equipped with a genetic machinery that allows it to survive in significantly different environments, allowing it to be of pest status on all continents except Antarctica [5,6,7]. The most widely used management strategy in the last 50 years has been the application of insecticides [3,4,7,8,9]. The short life cycle of DBM and a heavy reliance on insecticides has led to an expanding list of populations resistant to organochlorines, organophosphates, carbamates, pyrethroids, microbial-origin molecules and insect-growth regulators, as well as to novel mechanism insecticides [8,10,11].

In the developing world, the appearance of resistant populations has created vicious cycles of frequent (usually weekly) insecticide applications coupled with an increasing dosage. These practices have led to further resistance [11,12]. Although vegetable brassicas have a 90-day crop cycle, many farmers in Southeast Asia spray 12–16 times per cycle [7]. In the Americas, the dependency on and the failure of insecticides for DBM control is apparent in many countries. At the turn of the century, 15–20 insecticide sprays per crop cycle were common [13]. Currently, 36 sprays against DBM per crop cycle (three applications/week) are used across Nicaragua [13]. In Honduras and Costa Rica, spray regimes of nine applications per crop cycle were reported in 1997, and by 1999, this was increased to 16 applications per crop cycle [6,14]. More than two decades later, farmers report the same or higher number of applications [6,13,15].

Alternatives for DBM management have been explored and the advantages and disadvantages of integrated pest management (IPM) for this pest have been extensively discussed [2,4,7,12,16,17,18,19,20,21,22]. Among these alternatives, several studies have shown that the use of pheromone-baited traps as means of monitoring and mass trapping are two reliable measures with demonstrated results [20,21,23,24,25]. In Indian cabbage, significantly lower damage, higher marketable yields and a significant increase in gross profits were obtained in plots with an IPM program. The program consisted of using biological control by parasitoids, predators, application of neem, *Bacillus thuringiensis* and the insecticide phosalone only when pheromone-baited monitoring traps captured more than eight males/trap/night [21]. Subsequently, another study showed higher marketable yields when the insecticide cartap hydrochloride was applied only when monitoring traps captured average numbers of DBM males above eight, twelve, and sixteen males per trap per night for cabbage, cauliflower (*Brassica oleracea* var. *botrytis*) and knol khol (*Brassica oleracea* var. *gongylodes*), respectively [24]. 

Also, when it comes to mass trapping, pioneering work in India further demonstrated that mass trapping of DBM in multiple locations yielded consistently higher marketable yields than control plots under farmer practices [23]. In successive work, other authors have also shown that mass trapping yielded cabbage with 51–74% less damage in sites with mass trapping in comparison to control plots during two crop cycles [25]. 

In theory, pheromone-based decision-making and mass trapping work should have rendered calendarized applications against DBM in brassicas obsolete. Despite the clear benefits from the use of pheromone-baited traps for DBM control, to say that their use has not been widely implemented would be an understatement. Several reports have attempted to explain the underlining reasons for the lack of adoption [26,27,28,29,30]. Prophylactic insecticide spraying, which is common in tropical countries, has been heavily promoted by the pesticide industry for decades. This accelerates the development of resistance and decreases populations of natural enemies, which lowers the effectiveness of biological control [29]. Even microbially-derived products such as *B. thuringiensis* and Spinosyns have been victims of their own success as their efficacy has caused farmers to overuse them in calendarized-spray regimes that select for resistant populations [30]. Farmers, many of whom are small stakeholders, lack knowledge of IPM alternatives, distrust new techniques and are generally averse to risk. Most believe that IPM practices represent higher-cost control options and come with increased chances of losses [6,14,15,16,30].

A first comparison of implementing mass trapping for the management of DBM was done in Costa Rica in 2020, showing significantly lower costs in comparison to calendarized applications [31]. As a continuation of this work, the current report demonstrates that using mass trapping with pheromone-baited traps in Costa Rican and Nicaraguan cabbage plantings leads to a reduction of insecticide applications and generally increases yields, savings and profits. We compared DBM populations observed in 13 cabbage farms spread among four different crop cycles. We used this data to compare the costs involved and calculate the net profit between the use of male monitoring and mass trapping of DBM as a means of DBM management in comparison to common farmer practices which consisted of calendarized pesticide sprays.

## 2. Materials and Methods

A total of nine plots from different cabbage plantations were evaluated with IPM of DBM through mass trapping with pheromone traps. Experiments took place along four crop cycles in two different countries and their results on DBM average captures, cabbage head damage and number of insecticide applications were compared with controls either evaluated simultaneously (four) or through historical reported data (two). Plots managed DBM either through calendarized insecticide applications, also considered as farmer current practices (FCP), or through integrated pest management (IPM), which meant mass trapping, monitoring and *ad libitum* insecticide sprays based on damage perception by the farmer. Details for each of the plots assessed are provided below (Table 1).

### 2.1. Crop Cycle I

A commercial cabbage farm located in Pacayas, Cartago, Costa Rica was selected. In a 0.5 ha plot, a mass trapping regime was set using 30 plastic gallon traps similar to those used for fall armyworm [32], but baited with the commercial female-produced sex pheromone lure attractive to male *P. xylostella* (P054-lure, ChemTica, Santo Domingo, Costa Rica) designed to last over a period of 90 days (full crop cycle in cabbage). Traps were uniformly distributed in a grid (~13 m between traps, at a density of 60 traps per hectare). In each trap, 300 mL of soapy water (1% laundry detergent) was added to act as drowning solution for the captured insects. Each week, 20 traps were considered as monitoring traps, the number of DBM males was counted and soapy water was changed if needed. Additionally, each week during six weeks of the crop cycle, 40 randomly selected plants within the plot were subjected to a DBM damage assessment according to a scale of damage that considers DBM holes in outer, lower and inner leaves as well as the marketability of the heads (Table 2) [33]. 

Published work recommends insecticide sprays only at the threshold of 8 males/trap/night in any plot [21,24]. However, the farmer agreed that rather than following his normal calendar-based application regime, he would apply insecticides based on his damage perception (*ad libitum*). A second plot of the same farm and with cabbages of similar age located 150 m from the mass trapping plot was considered as a control plot or FCP plot (insecticide regime followed a calendarized basis). In the FCP plot, although DBM population was not monitored, plant damage assessment was performed weekly. For both the IPM plot and the FCP plot, the farmer recorded and reported the number of insecticide applications against DBM and the costs and yield per plot. 

### 2.2. Crop Cycle II

Within a commercial cabbage farm located in Cipreses, Cartago, Costa Rica, a newly planted 0.8 ha plot was utilized as an IPM plot. This plot received 48 pheromone-baited traps (approximately 12 m between traps at a density of 60 traps per hectare) and non-calendarized insecticide applications *ad libitum* responding to the farmers’ perception of damage. As in crop cycle I, captures in traps were evaluated weekly and soapy water was renewed whenever needed. No FCP plot was available but the farmer provided the information on average costs and expected yields normally obtained with his calendarized pesticide applications in a hectare of cabbage of his own (coded FCP2). 

### 2.3. Crop Cycle III

Six commercial cabbage plots were selected. The plots belonged to farmers near the localities of Capellades and Pacayas of Cartago, Costa Rica. Three plots of 3710, 3500 and 7000 m^2^ were used as IPM plots, each receiving the equivalent density of 60 traps/ha of pheromone-baited traps (approximately 13 m between traps). The other three plots corresponded to FCP plots and they were the property of the same farmers, but located 2–4 km away from IPM plots with the same cabbage age and variety. For each farm, three to six traps were randomly selected as monitoring traps and checked weekly. Soapy water was renewed for all traps every three to four weeks. For IPM plots, the spraying of insecticides against DBM was done by the farmer *ad libitum* but weighing the monitoring trap-derived information and also his perception of damage. FCP plots underwent calendarized insecticide applications. Captures from monitoring traps were recorded weekly during the crop cycle. For plant damage assessment, the same scale used during cycle I was employed utilizing 20 randomly selected plants in each plot. Farmers recorded and reported the number of insecticide applications against DBM and the costs and yields per plot.

### 2.4. Crop Cycle IV 

Four Brassicaceae-producing commercial farms in Jinotega and Estelí, Nicaragua were selected. At each farm, an area of 0.7 ha received a regime of 20 pheromone-baited traps with soapy water (approximately 26 m between traps, at a density of 30 traps per hectare). The number of males captured in each trap was recorded weekly for 5 weeks and application of insecticides was *ad libitum* following the grower’s perception of damage. All plots were treated equally when insecticide applications were needed. Costs from IPM and FCP plots were reported by the farmers.

### 2.5. Analysis

Average captures in pheromone-baited monitoring traps were assumed to be indicative of the relative populations in each plot. For damage assessment analysis, comparison of damage was only considered for those cases in which the damage score was above 3 according to the scale [33]. Damage scores below 3 do not lead to rejection by farm gate purchasers and therefore do no lead to unmarketable cabbage. The percentage of plants that had a score of 3 or higher at any point during the weeks of evaluation was determined for each farm in crop cycles I and III, and the averages of each pair of IPM and FCM plots were compared with a Z proportion test in R. Costs of DBM management (pheromones, traps, insecticides, labor) and yields were calculated using the information provided by the farmers whenever possible; otherwise, yield information obtained from reports issued by government offices were used. Gross profit was calculated from the price per ton paid in the national market for 2020 in Costa Rica (USD 490/ton) and Nicaragua (USD 150/ton) [34,35]. Net profit was calculated by subtracting the costs of DBM management (pheromones, traps, insecticides, labor) from the calculated gross profit corresponding to the obtained yields. In the case of the plots of farms of cycle IV, cost analysis is presented only for one scenario as in all four farms the pesticide spraying regime was reportedly the same.

## 3. Results

### 3.1. DBM Captures

**Figure 1:**
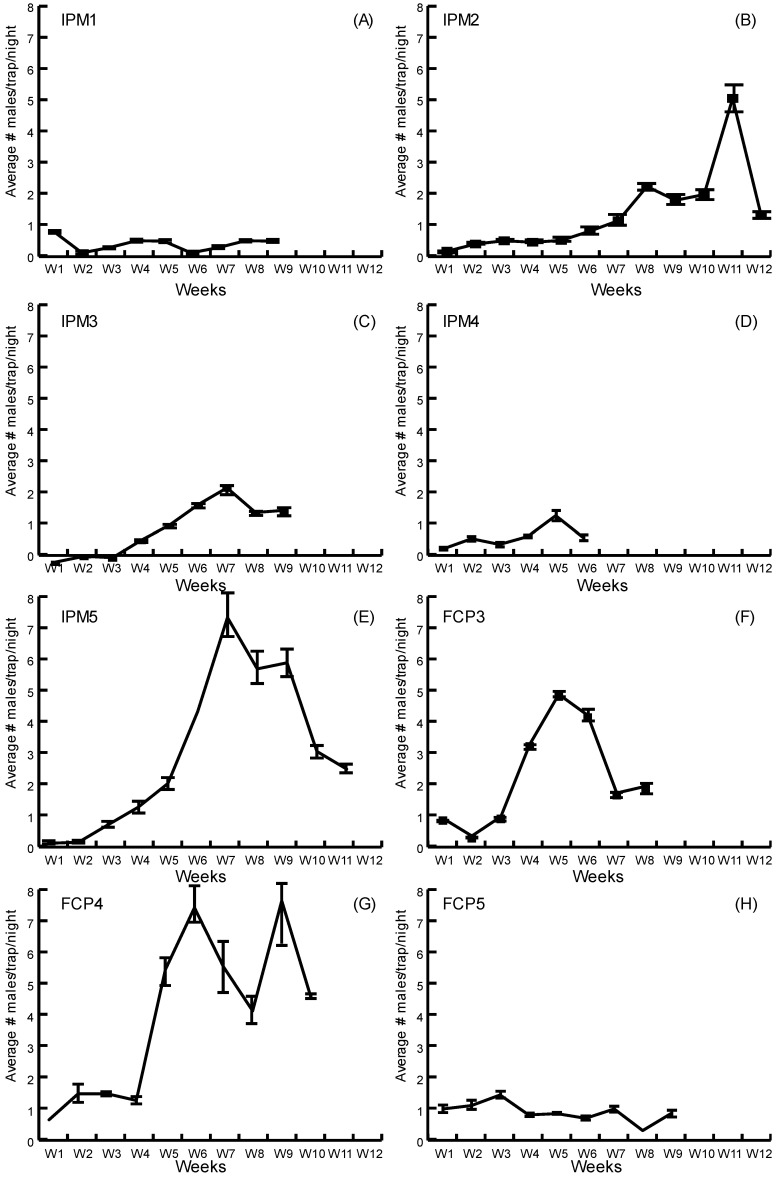
Average DBM male captures in IPM plots (**A**–**E**) and FCP plots (**F**–**H**) in Costa Rica. Standard error bars are shown for each evaluation.

Population levels observed with monitoring pheromone traps during cycles I and II evidenced numbers well below the established threshold of 8 males/trap/night throughout the whole crop cycle (Figure 1A,B). Although farmers continued to apply insecticide against DBM in IPM plots, a reduction of 20% for cycle I and 40% for cycle II occurred in comparison to the number of applications (10) made in FCP plots (Supplementary Tables S1 and S2).

During cycle III (Figure 1C–E), population levels were higher than in the previous two crop cycles, but only once did the average captures of males per night (IPM5, week 7, 7.24 males/trap/night) approach the threshold needed to trigger a spray. There was a reduction of 50%, 30% and 10% in the number of applications for IPM plots 3, 4 and 5, respectively (Supplementary Table S3). FCP plots (Figure 1F–H) showed trends similar to those in the IPM plots, but at one of the farms, populations were close or above the threshold on at least two occasions (FCP4, weeks 6 and 9, Figure 1G). These farms followed the protocol of weekly insecticide applications up to one or two weeks before harvest, which amounted to ten insecticide sprays in the crop cycle.

**Figure 2:**
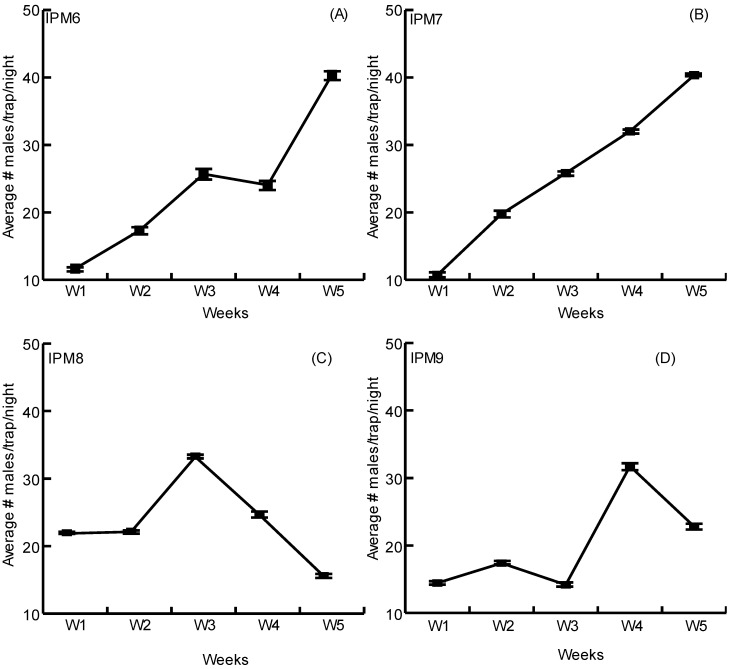
Average DBM male captures in IPM plots of four farms in Nicaragua (**A**–**D**). Standard error bars are shown for each evaluation.

Cycle IV farms in Nicaragua exhibited much higher populations than observed in Costa Rica (Figure 2). At every evaluation, the average numbers of male DBM captured in pheromone-baited traps were above the threshold of 8 males/trap/night, indicating the need for insecticide application. Interestingly, in Nicaragua, the farmers considered the damage and general populations to be lower than in unmonitored FCP plots. Therefore, the number of pesticide applications for the control of DBM in IPM plots decreased from 36 to 13 in each plot (Supplementary Table S4).

### 3.2. Cabbage Damage Evaluation

**Figure 3:**
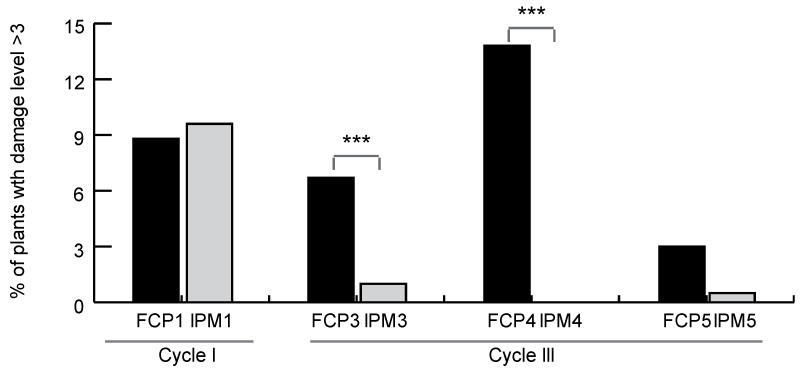
Percentage of damaged plants in plots with different DBM management strategies. Asterisks (***) denote significant differences between paired treatments (Z proportion test, *p* < 0.05).

Damage assessment revealed that in cycles I and III, only 8.9% of the cabbage plants had leaf damage above a score of 3 or were somehow unmarketable (score 5). Of those, FCP3 and FCP4 plots showed significantly higher percentages of cabbages with damage than their corresponding IPM plots (Figure 3).

### 3.3. Economic Analysis

Economic examination showed wide differences in the costs of insecticide applications, yields and gross and net profits among the different plots in the crop cycles evaluated within Costa Rica and when compared to Nicaragua (Table 3 and Table 4).

The farmer in charge of IPM1 and FCP1 during cycle I reported that no difference was observed in yields between methods, leading to an equal gross profit between the treatments. However, his perception of lower damage in the IPM plot allowed him to save at least three insecticide sprays, which lowered his expenses by at least USD 98 in the plot (i.e., USD 196 per hectare), which translated into a 1.96% higher net profit for the IPM plot. 

In cycle II, the farmer reported a higher yield in the IPM plot in comparison to the yields he normally obtained under the regime of calendarized insecticide applications against DBM. His net profit increased by approximately 20% as a result of higher yield and omission of at least three insecticide sprays.

There were some important differences among the farmers of cycle III. Plots under management of farmer 3 (plots IPM3 and FCP3) received about half the insecticide sprays of the IPM plot. However, the topography and location of the farm meant more labor and movement of machinery for each insecticide application and consequently increasing costs. Therefore, savings on insecticide application were not as pronounced. In addition, yields from IPM3 were much lower than from FCM3. The reason for this was not related to DBM but to the mismanagement of the crop in the IPM plot by the farmer. The IPM3 plot was located in land newly rented by the farmer who was testing different agronomical practices on the IPM3 plot while the trapping project was occurring. 

Plots under management of farmer 4 (IPM4 and FCP4) also presented striking differences between the two plots under study, but in this case in favor of trapping. An increase in yield and a decrease of at least three insecticide applications returned a net profit about 48% higher for the IPM plot. Finally, plots under management of farmer 5 (IPM5 and FCP5) received only one application less in the IPM plot that in the FCP plot. Since yields were higher in the IPM plot than in the FCP plot, net profit in the IPM plot was 8% higher than in the FCP plot. All farms were evaluated at the same time, were geographically close and contextually similar. We therefore averaged the net profits for all three farms to obtain a difference of about 11% in favor of trapping and IPM over FCP.

Cycle IV data on yields were obtained from national databases and not from Nicaraguan farmers. Our results indicate a reduction of insecticide expenditures to one third of what is usually applied (Table 3). Assuming that yields were at least the national average, i.e., 58 tons/ha, and sold at a farmer-reported price of USD 150 per ton, the net profit from IPM plots would have been USD 7486 per hectare. This value would be greater than 8% higher than the profit in FCP plots (USD 6880). Frequency of application and type of insecticides varied among IPM plots and FCP plots. Insecticide applications in FCP plots took place every three days with an arsenal of eleven rotated insecticides; insecticide sprays in IPM plots took place every seven days with a battery of eight insecticides. For the insecticides left out in IPM plots, see Supplementary Table S4. 

## 4. Discussion

Expenditure in DBM management is high due to the frequency of insecticide applications. An estimated USD 4–5 billion were spent worldwide in 2012 [3]. As previous and the present study show, in most locations, population levels often do not justify weekly insecticide sprays, much less three times within a week, as practiced in Nicaragua.

Scheduled-spraying of insecticides against DBM in cabbage-producing farms in Costa Rica and Nicaragua has been the default method of management for decades and most farmers have not been keen to change it. Within Costa Rican farms (cycles I-III, Figure 1), we found that except for very few cases, pheromone-baited trap captures did not surpass the threshold of eight males/trap/night used as a trigger to apply an insecticide spray [21]. Farmers in this study usually did not use this threshold as indicator and preferred to follow their own perception of plant damage. Interestingly, this deviation from calendarized insecticide sprays allowed Costa Rican farmers that were willing to trust the pheromone monitoring system to save an average of three insecticide applications per crop cycle. In Nicaragua, the populations were significantly higher (Figure 2) and at all times indicated the need for insecticide intervention [21,24]. Nevertheless, the farmers had a different attitude about what they were observing in plots with pheromone-baited traps. In their perception, the number of moths captured in pheromone-baited traps was high but assessment of crop damage did not indicate to them that there was a high infestation in the plots with traps. This allowed Nicaraguan farmers to reduce insecticide spraying to once a week, which is one third of what is normally applied in FCP plots. Opting to follow a management plan wherein insecticide applications occur only when the crop actually requires it is not a new idea. The benefits of such management strategies have been demonstrated in Australia, China, Korea and the South Pacific [3,4,29,30].

FCP plots presented significantly higher percentages of cabbage plants with damage levels above the threshold score of 3, which suggested the cabbages had to be at least trimmed to be marketable (Figure 3). Similar results were observed in two previously mentioned studies in India [21,24]. These studies observed significantly lower numbers of DBM larvae and percentages of plants with holes in leaves in sites managed with pheromone-baited traps versus control plots in which regular calendarized insecticide applications were made.

Insecticide expenditure was very different between DBM management methods as the costs of insecticide applications was always lower in the IPM plots (Table 3). Economic analysis (Table 4) showed that except for one case, the IPM plots produced higher net profits than the FCP plots. The case in which the yield of the IPM plot was lower than the FCP plot (farmer 3) was due to the external factor of additional testing that was occurring in the test plots.

Overall, Costa Rican farms experienced higher yields and lower insecticide costs that averaged to an increase in net profit of USD 1723/ha. In Nicaraguan farms, the putative average increase in net profit was USD 605/ha due primarily to a dramatic reduction in insecticide application. 

The direct impact on profit by reduction of insecticide applications has been mathematically modelled and demonstrated in practice since 2012, when Zalucki et al. re-calculated the worldwide costs of DBM management. This work demonstrated that weekly insecticide applications at the global scale were about four times more expensive than the use of IPM strategies. More recently, a Chinese study wherein insecticide sprays against DBM triggered by a threshold of more than 10 L1 larvae per plant showed that insecticide expenditure was cut by half [12].

The results observed in Nicaragua are especially noteworthy and representative of farmer’s attitude. In these farms, not only were the number of applications decreased to one third, but also the type of insecticides changed (Supplementary Table S4). Although DBM populations in Nicaragua are known to be resistant to *B. thuringiensis*, farmers were using this insecticide to manage DBM [6]. Those participating in the pheromone trapping trial abandoned this insecticide during the test. This may imply that the farmers probably knew of the low efficacy of the *B. thuringiensis* products but still used them as part of the “package” they are accustomed to spraying. 

There is an astounding difference between the population levels of DBM in cabbage production in Costa Rica and Nicaragua, which may be related to the relationship of farmers with insecticides. Insecticide applications in the cabbage farms studied in Nicaragua were significantly higher than in Costa Rica. At Nicaraguan application rates, populations of natural enemies would be more severely depressed. Although it was not used as a parameter in this study, we observed the presence of natural enemies in plots with traps in Costa Rica. Presently in Costa Rica, most cabbage farmers utilize three insecticides of relatively low environmental impact (Spinetoram, *B. thuringiensis* and Emamectin benzoate). It would be interesting to see if natural biological control in Nicaragua would benefit by using these and similar insecticides but less frequently than is currently practiced. It is also possible that DBM populations could be lowered in Nicaragua if farmers would embrace the recommended dose of 60 traps/ha for mass trapping rather than the density of 30 traps/ha used in this study.

The outcomes of our study add to several different demonstrations of DBM management through pheromone-baited traps [20,21,23,24,25]. The specific acting mechanism for population suppression of mass trapping is assumed to be the annihilation of males, rendering females with less opportunities for mating encounters and hence decreasing infestations. This phenomenon has been observed in other lepidopteran pests such as *Prays citri*, *Phtorimaea operculella* and *Tuta absoluta* [36,37,38]. 

Our results confirm in two Central American countries that strategies to apply insecticides against DBM only when needed, aided by pheromone-baited traps, lowers insecticide sprays and can increase profits to farmers. We firmly believe that economic benefit will be a strong driver of the adoption of any new technology in the farming sector.

## Figures and Tables

**Table 1:** Details of crop cycles, farms and management of each case analyzed.

Cycle	Plot Code	Size (ha)	Location	DBM Management	Number of Monitoring Traps	Number of Traps for Mass Trapping
I	IPM1	0.5	Pacayas, Costa Rica	Mass trapping + ad libitum insecticide sprays	20	30
	FCP1	0.5	Pacayas, Costa Rica	Calendarized insecticide sprays (10 per cycle)	-	-
II	IPM2	0.8	Cipreses, Costa Rica	Mass trapping + ad libitum insecticide sprays	20	48
	FCP2 *	1	Cipreses, Costa Rica	Calendarized insecticide sprays (10 per cycle)	-	-
III	IPM3	0.7	Capellades, Costa Rica	Mass trapping + ad libitum insecticide sprays	6	42
	FCP3	0.3	Capellades, Costa Rica	Calendarized insecticide sprays (10 per cycle)	3	-
	IPM4	0.7	Capellades, Costa Rica	Mass trapping + ad libitum insecticide sprays	6	42
	FCP4	0.7	Capellades, Costa Rica	Calendarized insecticide sprays (10 per cycle)	6	-
	IPM5	0.4	Pacayas, Costa Rica	Mass trapping + ad libitum insecticide sprays	6	23
	FCP5	0.3	Pacayas, Costa Rica	Calendarized insecticide sprays (10 per cycle)	3	-
IV	IPM6	0.7	Jinotega, Nicaragua	Mass trapping + ad libitum insecticide sprays	20	30
	IPM7	0.7	Jinotega, Nicaragua	Mass trapping + ad libitum insecticide sprays	20	30
	IPM8	0.7	Estelí, Nicaragua	Mass trapping + ad libitum insecticide sprays	20	30
	IPM9	0.7	Estelí, Nicaragua	Mass trapping + ad libitum insecticide sprays	20	30
	FCP6 *	1	Jinotega & Estelí, Nicaragua	Calendarized insecticide sprays (36 per cycle)	-	-

* Asterisks indicate that data was provided from the historical account of the farmers or by comparison with the current standard management reported by the farmers for one hectare of cabbage.

**Table 2:** Damage assessment scale.

Score	Description
1	No damage, few holes in outer or lower leaves
2	Many holes but limited to outer or lower leaves
3	Considerable damage in outer or lower leaves with some damage to head, marketable after removal of damaged leaves
4	Outer or lower leaves destroyed, considerable head damage, marketable after significant removal of damaged leaves
5	Severe attack of the head leading to unmarketable product

**Table 3:** Cost of DBM management per plot.

Plot Code	Cost per Insecticide Application (USD)	Number of Insecticide Applications	Total Cost of Insecticide Applications (USD, incl. Labor, Machinery, Insecticide Cost)	Total Cost of Mass Trapping (USD, incl. Pheromone, Traps, Labor)	Total Cost in DBM Management (USD)
IPM1	80	7	560	142	702
FCP1	80	10	800	0	800
IPM2	80	7	560	230	790
FCP2 *	80	10	800	0	800
IPM3	208	5	1039	64	1103
FCP3	128	10	1280	0	1280
IPM4	341	7	2387	128	2515
FCP4	305	10	3054	0	3054
IPM5	113	9	1022	66	1088
FCP5	109	10	1090	0	1090
IPM6-IPM9	50	13	650	200	850
FCP6 *	35	36	1260	0	1260

* Asterisks indicate that data was provided from the historical account of the farmers or by comparison with the current standard management reported by the farmers for one hectare of cabbage.

**Table 4:** Economic analysis of yields and profits from Costa Rican cabbage plots under different DBM management regimes.

Indicator	IPM1	FCP1	IPM2	FCP2	IPM3	FCP3	IPM4	FCP4	IPM5	FCP5
Expected yield (tons/ha) *	33	33	33	33	33	33	33	33	33	33
Obtained yield (tons/ha)	28	28	37	30	22	37	44	28	39	38
Gross profit (USD/ha)	13,720	13,720	18,208	14,700	10,927	18,228	21,560	13,720	19,355	19,012
Net profit (USD/ha)	12,220	12,120	17,220	13,700	9351	14,453	17,966	9357	16,422	15,019

* Asterisks indicate that data was obtained from the average reported at the national level by the Ministry of Agriculture authorities for the year 2020 [1].

## Data Availability

Available in supplementary tables.

## Supplementary Materials

The following supporting information can be downloaded at: https://www.mdpi.com/article/10.3390/insects14020149/s1, Table S1. Populations, Damage and Cost Analysis Cycle I, Table S2. Populations, Damage and Cost Analysis Cycle II, Table S3. Populations, Damage and Cost Analysis Cycle III, Table S4. Populations, Damage and Cost Analysis Cycle IV.

## References

[1] Food and Agriculture Organization of the United Nations FAOSTAT Statistical Database. https://www.fao.org/faostat/en/.

[2] Talekar N.S., Shelton A.M. (1993). Biology, ecology, and management of the Diamondback Moth. Annu. Rev. Entomol..

[3] Zalucki M.P., Shabbir A., Silva R., Adamson D., Shu-Sheng L., Furlong M.J. (2012). Estimating the economic cost of one of the world’s major insect pests, *Plutella xylostella* (Lepidoptera: Plutellidae): Just how long is a piece of string?. J. Econ. Entomol..

[4] Li Z., Feng X., Liu S.-S., You M., Furlong M.J. (2016). Biology, ecology, and management of the Diamondback Moth in China. Annu. Rev. Entomol..

[5] Zhou H., Lei G., Chen Y., You M., You S. (2022). PxTret1-like affects the temperature adaptability of a cosmopolitan pest by altering trehalose tissue distribution. Int. J. Mol. Sci..

[6] Perez C.J., Shelton A.M. (1997). Resistance of *Plutella xylostella* (Lepidoptera: Plutellidae) to *Bacillus Thuringiensis Berliner* in Central America. J. Econ. Entomol..

[7] Sarfraz M., Keddie B.A. (2005). Conserving the efficacy of insecticides against *Plutella xylostella* (L.) (Lep., Plutellidae). J. Entomol. Nematol..

[8] Banazeer A., Afzal M.B.S., Hassan S., Ijaz M., Shad S.A., Serrão J.E. (2022). Status of insecticide resistance in *Plutella xylostella* (Linnaeus) (Lepidoptera: Plutellidae) from 1997 to 2019: Cross-resistance, genetics, biological costs, underlying mechanisms, and implications for management. Phytoparasitica.

[9] Agboyi L.K., Ketoh G.K., Martin T., Glitho I.A., Tamò M. (2016). Pesticide resistance in *Plutella xylostella* (Lepidoptera: Plutellidae) populations from Togo and Benin. Int. J. Trop. Insect. Sci..

[10] Jiang H., Wenjun W.U. (2003). Advance of studies on insecticide resistance to Diamondback Moth (*Plutella xylostella* L.). J. Guizhou Univ. Nat. Sci..

[11] Hama H. (1990). Insecticide resistance of Diamondback Moth, *Plutella xylostella* in Japan. Japan Agric. Res. Quarter..

[12] Li Z., Furlong M.J., Yonow T., Kriticos D.J., Bao H., Yin F., Lin Q., Feng X., Zalucki M.P. (2019). Management and population dynamics of Diamondback Moth (*Plutella xylostella*): Planting regimes, crop hygiene, biological control and timing of interventions. Bull. Entomol. Res..

[13] Pérez C.J., Alvarado P., Narváez C., Miranda F., Hernández L., Vanegas H., Hruska A., Shelton A.M. (2000). Assessment of insecticide resistance in five insect pests attacking field and vegetable crops in Nicaragua. J. Econ. Entomol..

[14] Araya L., Monge L., Carazo E., Cartín V. (1999). Diagnóstico del uso de insecticidas para el combate de *Plutella xylostella* en Costa Rica. Manejo Integrado de Plagas.

[15] Nunez-Chacon M. Un 65% de Los Alimentos Que se Consumen en el País Contiene Residuos de Agroquímicos. https://semanariouniversidad.com/pais/un-65-de-los-alimentos-que-se-consumen-en-el-pais-contiene-residuos-de-agroquimicos.

[16] Machekano H., Mvumi B., Nyamukondiwa C. (2017). Diamondback Moth, *Plutella xylostella* (L.) in Southern Africa: Research trends, challenges and insights on sustainable management options. Sustainability.

[17] Shakeel M., Farooq M., Nasim W., Akram W., Khan F.Z.A., Jaleel W., Zhu X., Yin H., Li S., Fahad S. (2017). Environment polluting conventional chemical control compared to an environmentally friendly IPM approach for control of Diamondback Moth, *Plutella xylostella* (L.), in China: A Review. Environ. Sci. Pol. Res..

[18] Baur M.E., Kaya H.K., Thurston G.S. (1995). Factors affecting entomopathogenic nematode infection of *Plutella xylostella* on a leaf surface. Entomol. Exp. Appl..

[19] Schuster D.J., Workman R.B., Chalfant R.B. (1984). Evaluation of a visual damage threshold for management of Lepidopterous larvae on cabbage with pyrethroid insecticides. J. Agric. Entomol..

[20] Reddy G.V. (2011). Comparative effect of integrated pest management and farmers standard pest control practice for managing insect pests on cabbage (*Brassica* Spp.). Pest. Manag. Sci..

[21] Reddy G.V., Guerrero A. (2000). Pheromone-based integrated pest management to control the Diamondback Moth *Plutella xylostella* in cabbage fields. Pest. Manag. Sci..

[22] Macharia I., Löhr B., de Groote H. (2005). Assessing the potential impact of biological control of *Plutella xylostella* (Diamondback Moth) in cabbage production in Kenya. J. Crop. Prot..

[23] Reddy G., Urs K. (1997). Mass trapping of Diamondback Moth *Plutella xylostella* in cabbage fields using synthetic pheromones. IPC.

[24] Reddy G., Guerrero A. (2001). Optimum timing of insecticide applications against Diamondback Moth *Plutella xylostella* in cole crops using threshold catches in sex pheromone traps. Pest Manag. Sci..

[25] Topagi S.C., Bhanu K.R.M., Kumar C.T.A. (2018). Mass trapping technique using pheromones: A standalone method for management of Diamondback Moth, *Plutella xylostella* (Linnaeus) (Plutellidae: Lepidoptera) in cabbage. Int. J. App. Sci. Eng..

[26] Lohr B., Kfir R., Kirk A., Bordat D. (2004). Diamondback Moth *Plutella xylostella* (L.) in Africa: A review with emphasis on biological control. Proceedings of the International Symposium Improving Biocontrol of Plutella Xylostella.

[27] Morallo-Rejesus B., Inocencio E.L., Eusebio J.E. (1996). Comparative effectiveness of IPM-DBM technology versus farmers’ control practice for Diamondback Moth, *Plutella xylostella* (L.) Control. Philippine Entomol..

[28] Tibugari H., Jowah P., Mandumbu R., Karavina C. (2012). Tackling Diamondback Moth *Plutella xylostella* (L.) resistance: A review on the current research on vegetable integrated pest management in Zimbabwe. Arch. Phytopathol. Pflanzenschutz.

[29] Furlong M.J., Wright D.J., Dosdall L.M. (2013). Diamondback Moth ecology and management: Problems, progress, and prospects. Annu. Rev. Entomol..

[30] Furlong M.J., Zu-Hua S., Yin-Quan L., Shi-Jian G., Yao-Bin L., Shu-Sheng L., Zalucki M.P. (2004). Experimental analysis of the influence of pest management practice on the efficacy of an endemic arthropod natural enemy complex of the Diamondback Moth. J. Econ. Entomol..

[31] González-Fuentes F., Narváez-Niño S., Rodríguez-Chinchilla C., Vargas-Martínez A., González-Herrera A. (2023). Trampeo masivo de *Plutella xylostella*: Una alternativa ambiental y económicamente beneficiosa para su control en Costa Rica. Revista Bionatura.

[32] Salazar-Blanco J.D., Cadet-Piedra E., González-Fuentes F. (2020). Monitoreo de *Spodoptera* Spp. en caña de azúcar: Uso de trampas con feromonas sexuales. Agron. Mesoam..

[33] Hasheela E.B.S., Nderitu J.H., Olubayo F.M., Kasina M. (2010). Evaluation of border crops against infestation and damage of cabbage by Diamondback Moth (*Plutella xylostella*). Tunis. J. Plant Prot..

[34] Asociación de Productores y Exportadores de Nicaragua Información de Precios de Mercado. https://apen.org.ni/informacion-de-precios-de-mercados/.

[35] Programa Integral de Mercadeo Agropecuario Boletín de Precios del Mayorista al Minorista. https://pima.go.cr/wp-content/uploads/2020/05/SIMM_BOLETIN_PRECIOS_20200501.pdf.

[36] Sternlicht M., Barzakay I., Tamim M. (1990). Management of *Prays citri* in lemon orchards by mass trapping of males. Entomol. Exp. Appl..

[37] Larraín P., Guillon M., Kalazich J., Graña F., Vásquez C. (2009). Effect of pheromone trap density on mass trapping of male potato tuber moth Phthorimaea operculella (Zeller)(Lepidoptera: Gelechiidae), and level of damage on potato tubers. Chil. J. Agric. Res..

[38] Lobos E., Occhionero M., Werenitzky D., Fernandez J., Gonzalez L.M., Rodríguez C., Calvo C., López G., Oelshchlager A.C. (2013). Optimization of a trap for *Tuta absoluta* Meyrick (Lepidoptera: Gelechiidae) and trials to determine the effectiveness of mass trapping. Neotrop Entomol..

